# An Investigation into Art Therapy Aided Health and Well-Being Research: A 75-Year Bibliometric Analysis

**DOI:** 10.3390/ijerph19010232

**Published:** 2021-12-26

**Authors:** Zhen Liu, Zulan Yang, Chang Xiao, Ke Zhang, Mohamed Osmani

**Affiliations:** 1 School of Design, South China University of Technology, Guangzhou 510006, China; liuzjames@scut.edu.cn (Z.L.); xiaocsd@scut.edu.cn (C.X.); kezh@scut.edu.cn (K.Z.); 2 School of Architecture, Building and Civil Engineering, Loughborough University, Loughborough LE11 3TU, UK; m.osmani@lboro.ac.uk

**Keywords:** art therapy, bibliometric analysis, co-occurrence analysis, health and well-being, therapeutic method, children, adolescent, elderly, psychotherapy, virtual reality

## Abstract

Considering the physical, and psychological impacts and challenges brought about the coronavirus disease 2019 (COVID-19), art therapy (AT) provides opportunities to promote human health and well-being. There are few systematic analysis studies in the fields of AT, which can provide content and direction for the potential value and impact of AT. Therefore, this paper aims to critically analyze the published work in the field of AT from the perspective of promoting health and well-being, and provides insights into current research status, hotspots, limitations, and future development trends of AT. This paper adopts a mixed method of quantitative and qualitative analysis including bibliometric analysis and keyword co-occurrence analysis. The results indicate that: (1) the current studies on AT are mostly related to research and therapeutic methods, types of AT, research populations and diseases, and evaluation of therapeutic effect of AT. The research method of AT mainly adopts qualitative research, among which creative arts therapy and group AT are common types of AT, and its main research populations are children, veterans, and adolescents. AT-aided diseases are trauma, depression, psychosis, dementia, and cancer. In addition, the therapeutic methods are mainly related to psychotherapy, drama, music, and dance/movement. Further, computer systems are an important evaluation tool in the research of AT; (2) the future development trend of AT-aided health and well-being based on research hotspots, could be focused on children, schizophrenia, well-being, mental health, palliative care, veterans, and the elderly within the context of addressing COVID-19 challenges; and (3) future AT-aided health and well-being could pay more attention to innovate and integrate the therapeutic methods of behavior, movement, and technology, such as virtual reality and remote supervision.

## 1. Introduction

In December 2019, a novel coronavirus disease 2019 (COVID-19) broke out in Wuhan, China. With the rapid spread of the new crown virus, the World Health Organization (WHO) declared the COVID-19 epidemic as a public health emergency of international concern on 30 January 2020 [1]. The spread of the COVID-19 across countries globally has brought unprecedented challenges to all walks of life, such as the international public health, trade, economic, and education systems, which influence the well-being of human society [2]. In addition, the COVID-19 has negatively affected the global service and manufacturing industry [3], and caused commercial stagnation, especially in the tourism, hotel, education, retail, and health sectors [4]. Further, in order to overcome this global threat, the Centers for Disease Control and Prevention of the WHO proposes to adopt preventive measures, such as social distancing, washing hands, and wearing masks, and isolation policy to reduce the spread of the COVID-19 [5]. However, the isolation policy that restricts people’s movement not only has a significant impact on the economy, environment, and society [6], but also increases the symptoms of people’s post-traumatic stress disorder (PTSD) and feelings of confusion, loneliness, boredom, and anger in the psychological field [7]. For example, under the influence of the epidemic, some children and adolescents have developed anxiety, depression, sleep and appetite disorders, and social difficulties [8], and the elderly become lonelier and more fearful [9]. In short, faced with the sudden COVID-19 and respective confinements and lockdowns, not only have society and the economy been affected, but for people across the world, noticeable impacts on people’s mental and physical health have been created. Mental health includes emotional, psychological, and social well-being, such as anxiety, life pressure, depression, and suicide. Hence, therapy methods provide mental health patients with tools to identify and solve problems that arise from life stressors.

Among the many non-drug treatments, art therapy (AT) is a comprehensive treatment that uses psychotherapy [10] and artistic creation processes [11], such as drama, music, dance movements, and drawing to promote health and well-being. In addition, AT is a non-verbal intervention [12,13,14], which can aid mental disorders affecting people across all ages through the intermediary of art [15,16]. AT, as an expression therapy, can bring many benefits, such as encouraging communication and expressing emotions [17], promoting emotional catharsis and experiencing positive emotions [18], increasing positive behaviors [19], and improving the quality of life of patients [20,21,22] and their self-esteem [23,24,25]. The art creation process of AT provides a medium through which different realities, attitudes, and feelings can be expressed, examined, and tried [26]. It enables individuals to use art to express themselves creatively [27]. Thus, AT plays a positive role in alleviating many mental and physical health conditions, such as depression [28,29,30], psychosis [31,32,33], PTSD [34,35,36], dementia [19,37,38], cancer [39,40,41], and diabetes [42,43,44]. 

AT is a valuable way of expression, and art shows a great promise in the direction of a sustainable future [45]. Art-based methods and aesthetics generate emotional connections in many fields, such as healthcare, which can bring sustainable value and results to individuals, organizations, and collectives [46]. In addition, the study of AT has potential value in solving the ongoing impact of the post-COVID-19 era [47,48]. In general, AT is an effective intervention to promote health in the context of the COVID-19 epidemic, and the use of art presents a potential link with social sustainable development. However, the most of systematic analysis of AT-aided health and well-being studies are focused on specific fields, such as AT interventions for cancer patients [49], AT support for people with psychosis [27], AT assistance for traumatized children [50,51], and visual AT for cognitive and psychological symptoms in the elderly [52], which provide only partial information of value for understanding the content structure and research hotspots of the field of AT-aided health and well-being studies. Therefore, the aim of this paper is to analyze the current research status, research hotspots, research deficiencies, and future development trends of AT-aided studies from the perspective of promoting health and well-being.

## 2. Methods

This paper adopts a mixed research method, which comprises a bibliometric analysis to quantitatively examine AT-aided health and well-being, identifying significant research structure and research topics, and a follow-up qualitative review to reveal the content of different AT research themes. In the quantitative data analysis, histograms, pie charts, and visual maps of keyword co-occurrence analysis in VOSviewer are used to analyze the current research status and research hotspots of AT. Bibliometric analysis is widely used to analyze the published literature in a particular field, which helps to evaluate the trend of research activities over time [53]. Although, due to the complexity of scientific development, the use of bibliometrics can only be a very rough measure of the law of scientific development, it can reveal the statistical association between keywords and trend topics [54]. Keywords can represent the core theme of article content [55]. According to the co-occurrence analysis of the frequency of keywords appearing in article, a brief cluster analysis can be performed [56], which can reveal statistical connections between individual topics [57]. In addition, VOSviewer is a free software tool that can be used to create and visualize bibliometric maps of scientific publications, authors, journals, countries, institutions, and keywords [58]. In the review articles related to AT, the research methods greatly include scope review [59], integrative review [60,61], meta-analysis [62,63], and quantitative analysis [64]. Further, there is a lack of research that uses the visualization of keyword co-occurrence analysis to summarize the categories and research hotspots of AT-aided health and well-being. Therefore, this paper uses visual maps of keyword co-occurrence analysis in VOSviewer to summarize the theme category and research hotspots of AT-aided health and well-being, and summarizes the actual application of AT-aided health and well-being on the basis of bibliometric analysis.

**Figure 1:**
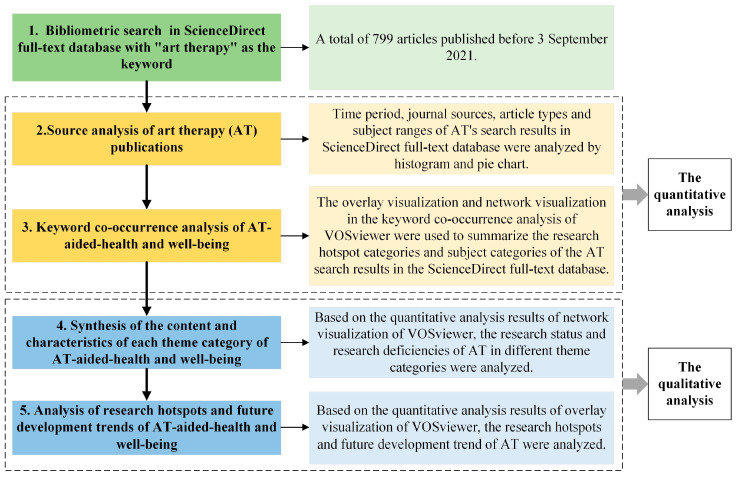
The flow chart of the research methodology.

ScienceDirect [65] is the full-text database platform of Elsevier, the world’s leading publishing company, and the world’s largest full-text electronic resource database for science, technology, and medicine. The resources on the ScienceDirect platform have established a broad, credible, high-quality interdisciplinary research and academic literature foundation from the four disciplines of physical sciences and engineering, life sciences, health sciences, social sciences, and humanities, which can help reveal the answers to the most urgent problems in the world. Therefore, this paper retrieves relevant data on AT-aided health and well-being from the ScienceDirect full-text database. As shown in Figure 1, the adopted research methodological flow chart shows five quantitative and qualitative data analysis steps: (1) bibliometric search in ScienceDirect full-text database with “art therapy” as the keyword; (2) source analysis of AT publications: using histograms and pie charts for data analysis; (3) keyword co-occurrence analysis of AT-aided health and well-being: using the visual map of keyword co-occurrence analysis in VOSviewer for data analysis; (4) synthesis of the content and characteristics of each theme category of AT-aided studies; and (5) analysis of research hotspots and future development trends of AT-aided studies. 

## 3. Results of the Quantitative Analysis of Art Therapy (AT)-Aided Health and Well-Being

### 3.1. Time Period Analysis

**Figure 2:**
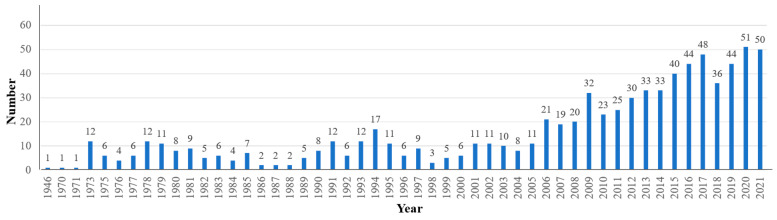
Distribution of articles published on art therapy (AT)-aided health and well-being in three quarters of a century, 75 years from year 1946 to September 2021 in ScienceDirect (devised by the authors).

In ScienceDirect full-text database, the research on the AT-aided health and well-being, which began in year 1946, encompasses a total of 799 published articles in three quarters of a century, spanning 75 years from 1946 to September 2021. These include including 191 articles from 1946 to 1999 and 608 articles from 2000 to 2021, which are shown in chronological order in Figure 2. In general, the number of the articles published on AT-aided health and well-being in ScienceDirect has shown an upward trend year-on-year. Since year 2006, the number of the articles ranges between 19 and 51 per year. In the recent two years (2020–2021), the number of the articles per year has reached the maximum so far, exceeding 50 articles, and is still increasing. This indicates that AT is gaining a continuous interest and widespread recognition among researchers. 

### 3.2. Journal Source Analysis

**Figure 3:**
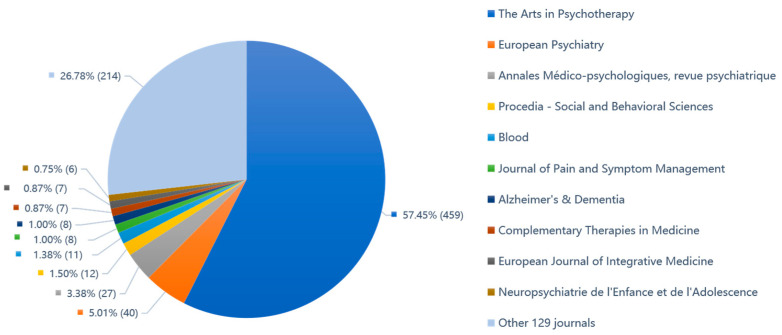
The journals for AT articles published in ScienceDirect (devised by the authors).

In ScienceDirect full-text database, the 799 articles on AT-aided health and well-being are published in 139 academic journals. As shown in Figure 3, the top five journals that publish most articles in the field of AT-aided health and well-being are ‘The Arts in Psychotherapy’ (formerly known as ‘Art Psychotherapy’), ‘European Psychiatry’, ‘Annales Médico-psychologiques, revue psychiatrique’, ‘Procedia-Social and Behavioral Sciences’, and ‘Blood’. That said, the AT-aided health and well-being articles were mainly published in the ‘The Arts in Psychotherapy’ contributing 459 articles, more than half of the total (57.45%), followed by ‘European Psychiatry’ journal (5.01%) with a total of 40 articles.

### 3.3. Article Type Analysis

**Figure 4:**
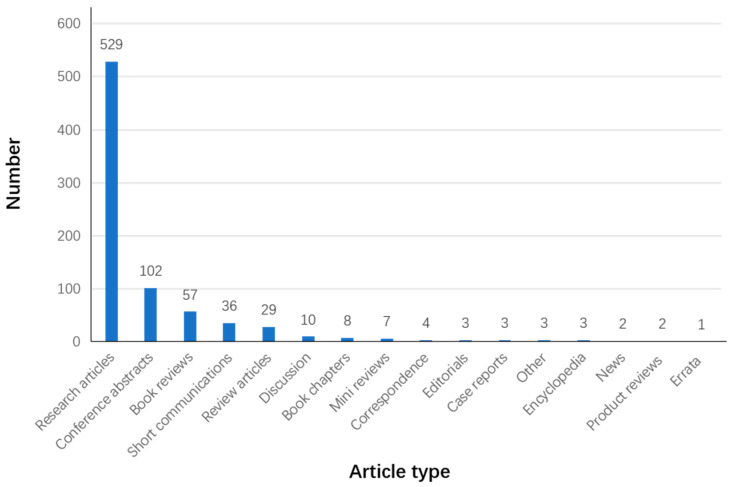
The number of different AT article types in ScienceDirect (devised by the authors).

As shown in Figure 4, the types of AT-aided health and well-being articles are principally research articles that have more than 500 articles, followed by conference abstract with 102 abstracts. In addition, there are 57 book reviews, 36 short communications, 29 review articles, and 10 discussions.

### 3.4. Keyword Analysis

#### 3.4.1. Overlay Visualization of AT-Aided Health and Well-Being

**Figure 5:**
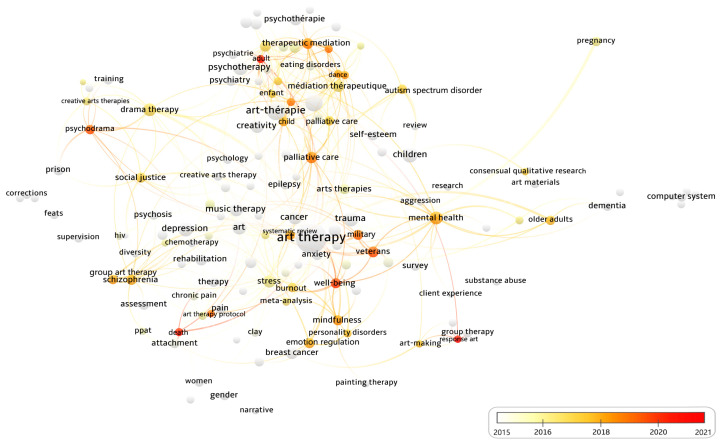
AT-aided health and well-being hotspots from 2015 to 2021 in the Overlay Visualization diagram generated by VOSviewer (devised by the authors).

The overlay visualization diagram generated by VOSviewer is shown in Figure 5, which illustrates the color of each keyword changes with the year, and the keywords in the red-orange-yellow circle are the research topics of the recent period. As indicated in Figure 2, the number of articles on AT has reached more than 35 and keeps rising since year 2015. Therefore, the period from recent years 2015 to 2021 is adopted for visual analysis of research hotspots on AT to illustrate current status and future directions. By and large, summarizing the red-orange-yellow keywords in Figure 5, and excluding keywords that are not related to the research topic, such as ‘burnout’ and ‘art therapy protocol’, the research hot keywords of AT-aided health and well-being in recent seven years (2015–2021) include five categories: (1) types of AT, e.g., group therapy and group AT; (2) therapeutic methods of AT, e.g., palliative care, mindfulness, dance, body image, memory consolidation, psychodrama, response art, therapeutic meditation, and emotion regulation; (3) research diseases of AT, e.g., schizophrenia; (4) research populations of AT, e.g., veterans, military, child, older adults, and adult; and (5) others: well-being, mental health, and death. It can be illustrated from the connecting relationship between the keywords of the research hotspot in Figure 5 that well-being and mental health are related to each other, where dementia to older adults is associated with mental health, and veterans and military are the main research groups for well-being subjects.

**Figure 6:**
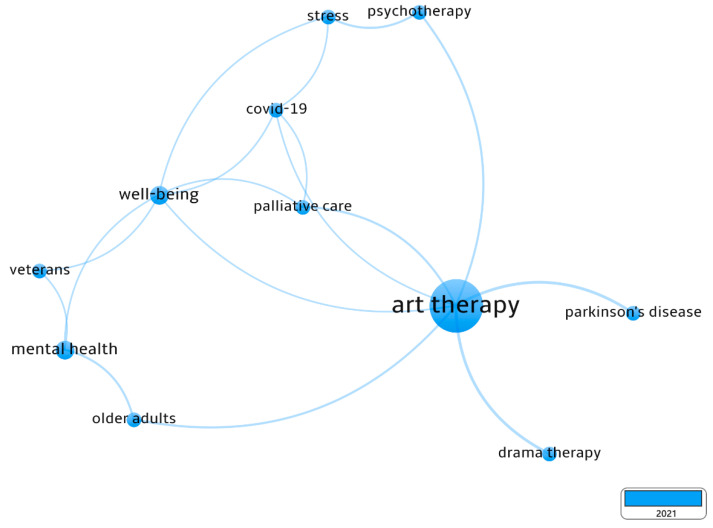
Latest research keywords of AT-aided health and well-being in 2021 via VOSviewer (devised by the authors).

As shown in Figure 6, well-being appears to be the most dominant AT topic in the latest research in 2021, indicating that AT involves more studies in well-being hotspots. In addition, the recent AT-aided well-being research topics are associated with mental health, COVID-19, palliative care, older adults, drama therapy, stress, psychotherapy, and Parkinson’s disease.

#### 3.4.2. Network Visualization of AT-Aided Health and Well-Being

**Figure 7:**
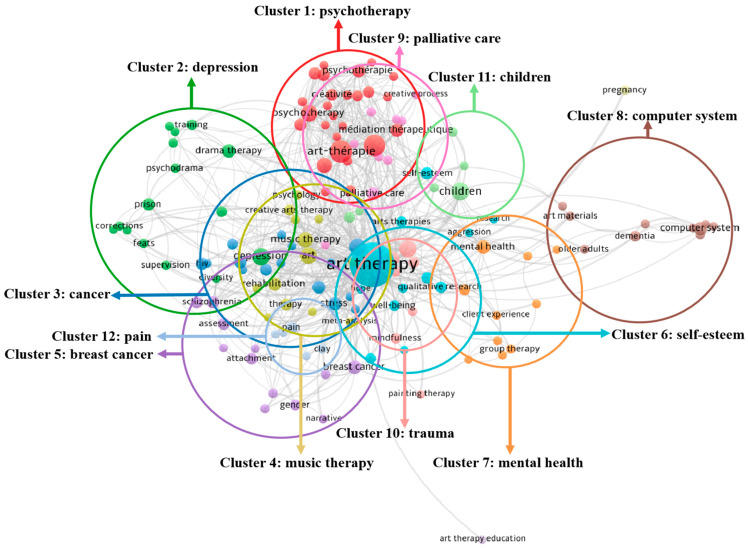
Twelve clusters of AT themes in the Network Visualization diagram generated by VOSviewer (devised by the authors).

In the Network Visualization diagram generated by VOSviewer, the more frequently keywords appear in the article, the larger the circle in the diagram and the more closely they are associated with the central theme. Since the VOSviewer software does not automatically merge the repeated synonymous keywords during the co-occurrence analysis of keywords, such as ‘art-therapy’, ‘art-thérapie’, and ‘art therapy’; ‘drama therapy’ and ‘psychodrama’; and ‘creative’ and ‘creativité’. Therefore, when classifying topics based on Network Visualization diagram, synonymous keywords are manually homogenized. It can be seen from Figure 7 that when AT is the central theme, there are several themes closely related to it, such as trauma, children, music therapy, depression, art, computer system, PTSD, drama therapy, assessment, creative arts therapy (CAT), dance/movement therapy (D/MT), prison, and breast cancer. In the keyword co-occurrence analysis of VOSviewer, circular nodes of different colors indicate different clusters. As shown in Figure 7, associated with the color change, all the keywords of the AT articles are divided into 12 clusters: psychotherapy; depression; cancer; music therapy; breast cancer; veterans; mental health; computer system; palliative care; trauma; children; and pain. 

In terms of the distance from each cluster to the center of AT in Figure 7, the cancer theme of the cluster 3 and the trauma theme of the cluster 10 are the closest themes to AT, indicating that the studies of AT have the closest relationships with trauma and cancer. On the contrary, the computer system of the cluster 8 is the furthest away from AT, which means that the subject of computer systems to aid AT needs more studies to bring them closer. In addition, in terms of the relative positions of the clusters in Figure 7, cluster 1 and cluster 9, cluster 3 and cluster 4, and cluster 6 and cluster 10 have an obvious intersection relationship, which reveals that psychotherapy and palliative care, cancer and music therapy, and trauma and veterans are closely related to each other. In terms of correlation within a cluster, the positions of CAT, D/MT, and music therapy are more concentrated in the cluster 4, indicating that the themes of this area are closely related.

Based on the keyword co-occurrence analysis results of VOSviewer, keywords that are most closely related to the AT theme are selected for further qualitative analysis in following Section 4. The co-occurrence frequency and total link strength of high-frequency keywords of AT-aided health and well-being are shown in Table 1.

Table 1 indicates that children (Occurrences: 20, Total link strength: 18), depression (Occurrences: 17, Total link strength: 17), and trauma (Occurrences: 17, Total link strength: 16) have a stronger co-occurrence relationship with AT-aided health and well-being. The AT articles employed primarily qualitative research methods, and the facilitated main diseases that the studies focus on are depression, trauma, and cancer. The treatment methods of AT are mainly related to psychotherapy, music, and drama. The population of AT-aided studies concern children, veterans, and adolescents. The reported benefits of AT are associated with improving the quality of life, mental health, self-esteem, and well-being. Moreover, CAT and group AT are the therapeutic categories of AT. Furthermore, the computer system is an important evaluation tool in the research of AT, which helps to further understand the value of AT in promoting health and well-being. Based on the content of Table 1, the existing research of AT-aided health and well-being are further classified by keywords in each cluster into six thematic categories, as shown in Table 2.

## 4. Results of the Qualitative Analysis of AT-Aided Health and Well-Being

Based on the results of the above quantitative analysis, this paper qualitatively summarizes the research status of AT-aided health and well-being in ScienceDirect full-text database from the following six thematic categories that are in line with Table 2: (1) research methods of AT-aided health and well-being; (2) types of AT; (3) research populations of AT; (4) AT-aided diseases; (5) therapeutic methods of AT; and (6) evaluation of therapeutic effect of AT-aided health and well-being.

### 4.1. Research Methods of AT-Aided Health and Well-Being

Qualitative research is the main research method of AT [66,67,68]. In qualitative research, the commonly used methods largely include semi-structured interviews [69,70,71], focus groups [66,72,73], phenomenological analysis [74,75,76], thematic analysis [67,77,78], and grounded theory [79,80,81]. Qualitative research can help with understanding the value of AT in terms of knowledge system, perception, experience, inner change, and perception. Smeijsters and Cleven [66] argue that qualitative investigation methods, such as semi-structured questionnaires, interviews, and focus groups, can describe the knowledge system of forensic psychiatric AT based on practice, theory, and research. Moreover, the qualitative research method of phenomenology can be used to understand the views of art therapists and patients on art creation and AT [69,82,83], and the theoretical framework of AT can also be understood from the experience of art therapists [76]. Through the qualitative research method of thematic analysis, the inner changes in patients during AT can be observed, and the inner changes in patients can be described through a common language [77]. In addition, thematic analysis of the results of interviews with professional therapists reveal that the new virtual reality (VR) medium has great potential in AT [84]. Furthermore, qualitative ground theory and interviews can assess the perceived effects of emotional management in patients with personality disorders to understand the effects of AT in clinical practice [80].

### 4.2. Types of AT

The types of AT-aided health and well-being mostly include CAT and group AT. CAT is a term used in therapeutic arts disciplines [64], which can use different forms of artistic expression such as music, drama, and dance/movement for treatment. Group AT, in which patients participate in the art centered treatment process, mainly includes five art therapeutic factors: symbolic expression; relational aesthetics; embodiment; pleasure/play; and ritual [85]. In group AT, mothers and children are the main research population.

#### 4.2.1. Creative Arts Therapy (CAT)

CAT has a certain potential in relieving psychological, behavioral, physiological, and other related diseases. It has been widely used to study military groups. Table 3 summarizes CAT studies in research methods, research populations, research diseases, and therapeutic methods. In CAT research, dance/movement, drama, and music therapy are often integrated [35,86,87]. Moreover, healing CAT from refugee backgrounds has shown a positive impact on young patient’s mood and behavior [86]. Research indicated that therapeutic value of CAT in treating traumatized children [51], and CAT have a positive impact on the psychology of patients with breast cancer and gynecological cancer [64]. CAT can also provide rehabilitation services for active military and veterans [88]. Further, telemedicine based on CAT provide healthcare opportunities for veterans [87,89]. In general, CAT help young people recognize and discover the meaning in their feelings; and help them improve their social and psychological functions [43].

#### 4.2.2. Group AT

Group AT has numerous benefits for mothers and children, such as relieving symptoms of depression, fear, anxiety and mood disorders, and improving patients’ subjective well-being and quality of life. Table 4 lists group AT studies in research methods, research populations, research diseases, and therapeutic methods. Choi and Goo [98] prove that group AT can effectively change the mother’s parenting behavior through a mixed research method including experiments, questionnaires, and case studies. For mothers, group AT is able to improve the mental health and well-being of mothers regarding their disabled children [99]. Similarly, for children, group AT improve the overall health-related quality of life of children with cancer [40]; and bring positive effects for children with attention deficit hyperactivity disorder (ADHD) and emotional dysregulation [100]. Moreover, group AT can not only reduce the depression level of elderly patients with neurocognitive disorders patients and improve their ability to express themselves [101], but also reduce the fear of childbirth in late pregnancy [102]. Furthermore, group AT facilitated with respiratory therapy allow anxious patients to recognize their negative emotions, which can reduce anxiety symptoms and improve subjective well-being [103].

### 4.3. Research Populations of AT

The research population of AT-aided health and well-being chiefly focusses on children, veterans, and adolescents.

#### 4.3.1. Children

AT-aided health and well-being with children as the research population largely adopt a mixed/hybrid research method integrating experiment, interview, questionnaire, and case studies [114,115,116]. AT tends to have a positive therapeutic effect on children’s behaviors, learning disabilities, trauma, autism, and other diseases. Indeed, AT can improve their academic performance [117], enhance emotional and behavioral adaptability [115], and reduce anger and improve self-esteem [24]. The integration of AT and music helps with developing and coordinating children’s personal abilities with special needs, which can help them integrate into society [118]. In addition, AT helps with reducing the trauma and stress symptoms of children [119], as well as improve the social/emotional skills of children with autism [120]. AT for improving self-image brings qualitative benefits to children with epilepsy [72] and autism spectrum disorder [116]. Further, drawing and painting is a commonly used form of AT-aided health and well-being for children because art therapists can understand brain activity in the process of drawing, which in turn, provides guidance for the theory and practice of AT [121]. Using drawing as a medium, AT improves understanding the personal experience of art therapists in treating aggressive children [122]. Drawing in a less threatening manner can relieve symptoms of ADHD and emotional dysregulation in children [100] and help children with epilepsy to express their feelings in non-verbal ways [123]. Using painting as an expression to explore emotions enable mother and child to solve depressed inner conflicts and reduce the fear of exposing oneself in the AT process [124].

#### 4.3.2. Veterans

The research of AT on the veterans group mainly adopts the case study method [12,36,125]. In the military population, PTSD and traumatic brain injury (TBI) are the most frequently interfered by AT. AT can help with understanding the inner experience of veterans with PTSD to promote trauma treatment [36]. Long-term AT is capable of improving the overall quality of life of soldiers [125], as well as satisfaction of PTSD and TBI patients with treatment and experience positive emotions, thereby reducing the symptoms of trauma [18]. AT is gradually being accepted as a form of care for military groups by helping senior soldiers with PTSD and TBI overcome psychological and physical symptoms [12]. With the development of technology, telemedicine based AT provides veterans with opportunities for mental health care and rehabilitation [87,89].

#### 4.3.3. Adolescents

The majority of AT-aided health and well-being on adolescents use case analysis research method [23,126,127], which largely discuss the therapeutic effect of AT on the neural, psychological, and behavioral problems of adolescents. In addition, group AT is a more effective form of treatment for adolescents [128]. When group AT is associated with respiratory therapy, it can stimulate the emotional and human characteristics of adolescents and significantly improve the subjective well-being of anxious adolescents [103]. Moreover, clay-based group AT has a positive impact on adolescents’ self-resilience [108]. CAT improves neurasthenic adolescents’ symptoms through stretching exercises and walking meditation [129]. Further, AT can help with reducing hopelessness, suicidal determination, and symptoms of PTSD for suicidal adolescents [23].

### 4.4. AT-Aided Diseases

AT-aided diseases are largely focused on five aspects: (1) trauma and PTSD; (2) depression; (3) schizophrenia and psychosis; (4) Alzheimer’s disease and dementia; and (5) cancer.

#### 4.4.1. Trauma and Post-Traumatic Stress Disorder (PTSD)

AT-aided health and well-being for trauma and PTSD mostly conducted via case study research method [12,14,125]. AT provides non-verbal treatment for trauma patients [14]. The scope of traumatic events mostly includes war, post-disaster, and displacement. The most common artistic method for treating trauma and PTSD is drawing, which is presented in different forms such as bridge, self-portrait, and mandala. Trauma is a non-verbal problem concept [130]. Non-verbal value can be discovered through artistic creation [12]. Hence, AT is an effective, non-verbal treatment method in solving trauma-related problems [14]. In the context of war, AT provides soldiers with an opportunity to explore their experiences, thoughts, and feelings through a nonverbal way [131]. The trauma children experience after a disaster can be treated with semi-structured artistic interventions [132]. Moreover, AT helps displaced people with strengthening their connection with society [128]. In the process of AT based on painting, the visual art form of bridge drawing can provide an AT opportunity for orphans who have experienced psychological trauma [133]. Among them, drawing in the form of self-portrait is able to reduce the impact of traumatic events [134]. Furthermore, the drawing form of the mandala is capable of facilitating the emotional catharsis of sex trafficking survivors and solve trauma problems [14].

#### 4.4.2. Depression

AT-aided health and well-being on depression primarily implement experimental research methods with scales [100,102,133], and clinical diagnostic methods [28,110,135]. AT can replace medical treatment to help relieve the symptoms of patients with depression and anxiety [136]. AT in the form of drawing improves depression in cancer patients [39]. In the experimental method, the evaluation with the Beck Depression Inventory (BDI) scale proves that AT is effective in reducing the depressive symptoms of male and female prisoners [137]. Group AT improves the depression condition among the elderly [101], and integrates respiratory therapy to heighten the well-being of anxious adolescents [103]. In addition, in the process of artistic creation, patients with depression recognize themselves through inner dialogue [138], and effectively alleviate various symptoms of patients with major depressive disorder through artistic psychotherapy [139]. Further, studies in clinical diagnosis highlight that AT is an effective psychotherapy method for depression [28], which has the ability to improve the quality of treatment and life of patients with depression [135].

#### 4.4.3. Schizophrenia and Psychosis

AT-aided studies principally employ case study research methods [140,141,142] and experiment [32,109,143] in investigating schizophrenia and psychosis. AT is one of the new techniques of human psychiatry, which can relieve and cure psychological disorders [144]. Using artistic creation as a medium may affect psychopathology and reduce the incidence of schizophrenia by strengthening the patient’s self-awareness [145]. When AT is facilitated with psychological education, psychiatric symptoms can be rapidly improved [141]. Paintings created by psychiatric patients provide an opportunity to perceive the patient’s inner world [140]. The intervention of AT in female patients with schizophrenia reduces symptoms and improves cognitive function [32]. However, there is no clear conclusion on the evidence of the efficacy and effectiveness of AT for patients with schizophrenia [146,147]. Although AT has uncertain evidence for the effectiveness of psychosis, many art therapists and psychiatric patients believe that AT is a beneficial, meaningful, and acceptable intervention [27].

#### 4.4.4. Alzheimer’s Disease and Dementia

Case studies were predominantly used in the AT-aided disease research to treat Alzheimer’s disease and dementia [20,148,149]. AT is part of non-drug treatment for patients with Alzheimer’s disease and dementia [20], which can relieve behavioral symptoms, and improve patients’ self-esteem, quality of life, and happiness [150]. In the AT-aided Alzheimer’s disease, a human-centered approach increases the convenience of the nursing process, in which empathy builds trust between the therapist and the patient [151]. Visiting museums and expressing AT improve the self-esteem and positive behavior of patients with Alzheimer’s disease [19]. In the care of dementia patients, participation in the intervention of AT enable dementia patients to alleviate the behavioral and psychological symptoms [37,150], improve the quality of life and well-being of patients [20], and may also help caregivers maintain health and improve well-being [148]. Hence, AT is seen as a valuable resource for solving mental health difficulties and/or challenges caused by dementia [38].

#### 4.4.5. Cancer

AT-aided cancer treatment studies have been conducted via a mixed research method using experiment, interview, and questionnaire [39,152,153]. Drawing is the most common form of AT to aid cancer patients. It has been reported that drawing activity in AT improves the mental health and quality of life of cancer chemotherapy patients [39,40,49]. In addition, the intervention of artistic creation can relieve the symptoms of cancer [154]; and is also beneficial to the stressful caregivers of cancer patients [18]. For cancer patients, AT is often associated with palliative care to reduce pain and symptoms [155], which improves the quality of life of the cancer patients [22].

Breast cancer is the disease that has been paid the most attention regarding the use of AT to treat cancer. The ‘Coping Resources Inventory’ is a commonly used assessment method, in which quality of life, fatigue, and subjective well-being are commonly used assessment elements in the process of AT treating breast cancer patients. It is able to explain and help with understanding the female image of breast cancer patients [156]; and prove that AT has important and positive significance in supporting health and coping resources in the short term [157]. In addition, the integration of cognitive behavioral intervention into AT can help the treatment of breast cancer patients. The artistic creation process of AT improves the quality of life [158], reduces fatigue symptoms [152], and enhances self-efficacy of breast cancer patients [159]. Reynolds and Lim [69] conducted a qualitative study revealing that artistic creation in the process of AT enables women with breast cancer to improve the subjective well-being and establish a positive life experience. Interestingly, art-based cognitive-behavioral therapy (CBT) was found to reduce anxiety and pain symptoms of breast cancer patients [160].

### 4.5. Therapeutic Methods of AT

The therapeutic methods of AT are twofold: psychology, and artistic creation. In addition to the drawing mentioned in Section 4.3.1 and Section 4.4.1, the therapeutic methods of AT-aided health and well-being include psychotherapy, drama therapy, music therapy, and D/MT.

#### 4.5.1. Psychotherapy

Psychotherapy is a biopsychosocial method in the process of AT [10]. Art psychotherapy brings positive changes to both staff and patients [161], such as promoting life coping skills, improving psycho-neural immune function, and enhancing interpersonal relationships [10]. There are various approaches of art psychotherapy, such as parent–child art psychotherapy, and mindful art psychotherapy. Parent–child art psychotherapy refers to paying attention to changes in the parent–child relationship in the presentation of art materials [162]. The use of parent–child art psychotherapy in the education system improves children’s emotional function, interpersonal relationship, and learning ability in a regular school environment [68]. The use of art in mindfulness-based psychotherapy can restore the cognitive reserve of the elderly, and improve mood and the pursuit of meaningful activities after retirement [163]. In addition, mindfulness art psychotherapy based on digital light therapy technology can help the elderly to externalize and express emotions; and improve symptoms of anxiety and depression [164]. The great potential of integrating technologies in art psychotherapy, such as VR, and light therapy, can accelerate and expand the process of AT. Using VR environment as a therapeutic environment in psychotherapy is able to bring possibilities for the innovation of artistic creation and constitute a new medium for AT [84]. 

#### 4.5.2. Drama Therapy

Drama is a friendly art form [165], as an additional embodiment of therapeutic artistic creation [97], associated with music therapy has a better therapeutic effect. Drama therapy facilitated with Interpretive Phenomenological Analysis (IPA) methods can effectively promote the treatment process. In the process of drama therapy, the integration of IPA method to analyze the experience of the creative art therapist is capable of alleviating the patient’s sense of self-insult, improve the therapist’s chances of understanding the patient, which contributes to the patient’s mental health [96]. In addition to understanding the experience of art therapists, IPA can also understand prisoners’ views on CAT in prison [82]. Moreover, the comprehensive psychological drama theory, and the model of CBT and narrative therapy (CBN Psychodrama) improve the self-control of high-risk adolescents [166]. The CAT-aided drama and music has the potential to reduce the symptoms of traumatic stress caused by school shootings in teenagers [35]. Furthermore, it was evidenced that drama therapy has a positive effect on severe mental patients [143], and has improved the emotional state of forensic patients [167].

#### 4.5.3. Music Therapy

Music therapy provides opportunities to treat behavioral, psychological, and cognitive disorders [168]. It is often associated with drama, dance/movement, and cognition-behavior to study the therapeutic effects of AT on different diseases. Music-based CAT can alleviate the emotional symptoms and behavioral problems of refugee youth [86], and may also be valuable in the treatment of drug abuse disorders [169]. Furthermore, CBT-based music improves anger management skills in forensic psychiatry [170]; and may also be an effective intervention for the treatment of fatigue in blood and bone marrow transplant patients [171]. Further, the integration of art and music therapy enable children with special problems to has a general, nonverbal advantage [119].

#### 4.5.4. Dance/Movement Therapy (D/MT)

D/MT is a new field of CAT [91], which largely focuses on mental illnesses, such as PTSD, mental disorders, emotions, and stress. It helps with exploring the psychological and behavioral changes in children suffering from PTSD after an earthquake; and providing cohesion for a group of children with mental disorders [172]. Additionally, D/MT has certain positive effects in reducing negative emotions [173] and alleviating stress problems [174]. It focuses on physiological diseases, such as breast cancer and nasopharyngeal carcinoma. D/MT for cancer patients can reduce their stress and improve their self-esteem [175]. In the disorder of behavior, the use of D/MT can deal with violence in prison [91]. Among the symptoms of perceived relief, D/MT utilizes video interpretation to help patients relieve pain symptoms [176]. Although D/MT has a positive therapeutic effect on many diseases, many articles indicate that a larger, complete, and blind random sample is needed for the specific discussion of D/MT [177,178,179].

### 4.6. Evaluation of Therapeutic Effect of AT-Aided Health and Well-Being

The computer system acts as an evaluation tool for AT-aided health and well-being. Table 5 summarizes the studies for computer system in the evaluation of therapeutic effect on AT-aided health and well-being. It can also be used as an auxiliary treatment tool for AT [180]. Color is a commonly used and effective evaluation index for the computer systems. By evaluating the colors in AT paintings, the effectiveness of the computer system can be verified [181], and it also provide useful information for the evaluation results of AT [182]. Among the various drawing forms evaluated by the computer system, mandala and Person Picking an Apple from a Tree (PPAT) are common methods. In addition, the stepwise regression model is a commonly used method for studies using computer systems. When using a stepwise regression model, multiple art treatment methods can be compared, among which PPAT is the best treatment method for patients with dementia [183]. In addition, the use of PPAT for evaluation also prove that human–machine evaluation tools may be more accurate than human perception [184]. A computer AT system for kinetic family drawing can bring objective and accurate evaluation for the evaluation of AT [185]. When using a computer system to evaluate the various elements of structured mandala, quantitative data could be generated to facilitate the decision-making process of experts [186].

## 5. Discussion

### 5.1. Research Hotspots and Development Trends of AT-Aided Health and Well-Being

Based on the quantitative and qualitative analysis results of this paper, it can be seen that group AT, children, and schizophrenia are the key themes of AT-aided health and well-being. There was consensus in the reviewed literature that AT is able to improve the quality of life and well-being of patients with Alzheimer’s disease, dementia, and breast cancer. Overall, the qualitative analysis results also show that well-being and mental health are closely related to AT.

The research hotspots of AT-aided studies from 2015 to 2021, primarily revolve around therapeutic methods and population. The four therapeutic methods of psychotherapy, drama, music, and D/MT all bring potential value to improve mood and psychological disorders. AT is also often associated with palliative care to explore the therapeutic effect. In addition to alleviating the symptoms of patients, AT-based palliative care interventions help patients and their relatives to improve their sensory, emotional, cognitive, and spiritual experiences [189], elevating the quality of life [22] and the effect of mood therapy [190]. Using dual AT between palliative care patients and their caregivers can protect the dignity of dying patients and their caregivers [191]. Kometiani and Farmer [14] believe that in the palliative care environment, using “representative art”, that is, art therapists to create works of art on behalf of patients, can facilitate the treatment of patients affected by fatigue and other serious diseases. 

Among the hot research population on AT, in addition to children, adolescents and veterans, the elderly has also become the key research population of AT. The positive therapeutic effect of AT on the elderly is mainly reflected in helping them prevent cognitive decline [192], and improving the ability of older survivors to share and process their stories and find meaning in life [193]. 

Furthermore, the results of AT-aided health and well-being indicate that among the latest research keywords in 2021, the research content of AT also revolves regarding the keyword of COVID-19. In the context of the COVID-19 pandemic, Hass-Cohen et al. [194] studied the effects of pain, depression, anxiety, interpersonal relationships, helplessness, and resources on patients participating in AT using drawing as a medium, before and after the pandemic, and found that the frequency of experiencing hopefulness of participants before the pandemic increased with time, while the frequency of experiencing hopefulness of participants after the pandemic decreased with time. From the perspective of research hotspots, the future development trend of AT may be based on research hotspots, such as group AT, children, schizophrenia, well-being, mental health, palliative care, veterans, and the elderly, for the COVID-19 pandemic associated topic.

### 5.2. New Therapeutic Methods for AT-Aided Health and Well-Being

The results reveal that the primary therapeutic methods of AT-aided health and well-being include psychotherapy, D/MT, drama, music, and drawing. Drawing includes self-portrait, mandala, PPAT, and bridge drawing as its approaches. In addition, AT-aided cognitive behavior is an effective method to promote health, which focuses on the therapeutic effect of facilitating behavior with psychodrama [166,195], and music [170,171]. CBT is considered a broad psychological approach [196], which helps to increase self-esteem [197] and reduce anxiety symptoms [198]. However, although CBT is a beneficial treatment method, the application of behavior as the CBT research theme with AT is few and new, which needs further investigation. In addition to CBT, D/MT has positive therapeutic value in the treatment of a number of diseases, such as autism, cancer, and PTSD. D/MT is a creative psychotherapy method based on the movement metaphor [199], whereby using the kinesthetic experience of movement metaphor, schizophrenic patients can express their emotions through words [200]. However, the specific effects of D/MT require more consideration, such as improving interpersonal competence [62], alleviating psychotic symptoms in patients with schizophrenia [173], and reducing emotional eating in obese women [201], which presents the status quo of insufficient research on D/MT. From the perspective of research deficiencies, there is a need for the future research of behavior and movement in AT-aided study towards promoting health and well-being, where the new therapeutic methods can be explored to enhance potential value of AT. 

### 5.3. Emerging Technology Enhanced AT-Aided Health and Well-Being

In the light of mega digital era, AT needs to utilize continuously evolving emerging technologies to make an effective intervention in its process. The application of emerging technology includes digital technology and remote technology, such as VR, digital phototherapy technology, computer technology, and telemedicine technology in the current state of AT-aided health and well-being. Mihailidis et al. [73] argue that the field of AT requires technical solutions to meet the needs of therapists and patients. Digital technology can be used for the training of art therapists to understand the relevant ethics of using digital media [202]. In the application of digital technology, computer technology is primarily used for the evaluation of the effect of AT, and VR is implemented to aid the process of AT. In addition, the experiment of professional art therapists using VR technology to create visual art shows that the VR medium has a great potential to facilitate process of AT [84]. Although the VR technology has shown a potential therapeutic value in delivering AT, there are insufficient studies on the application of VR technology to aid AT treatment, which is still in its infancy. Compared with digital technology, remote technology is mostly used in the rehabilitation and health care services of veterans [87]. However, remote supervision is mostly conducted in general counseling and education fields, with few applications in the field of AT [203]. Interestingly, among the various technologies, only digital technology and remote technology are commonly used in AT. By and large, while emerging technologies have enhanced AT for health and well-being, there are insufficient studies in the field of AT that integrate digital technology and remote technology for therapeutic intervention in neither methods nor approaches.

## 6. Conclusions

With the use of mixed research method, this paper summarizes the current situation, hot spots, deficiencies, and future research trends of the practical application of AT from both quantitative and qualitative aspects in promoting health and well-being, which provides specific content and direction for the potential practical value of AT. This paper has three main contributions: (1) this paper is the first mixed research method to incorporate AT articles of 75 years (3/4 century) by using visual keyword co-occurrence. This comprehensive research result has reference value for AT researchers, educators, and healthcare practitioners and can provide pathways for information and communications technology (ICT) development for information visualization software suppliers. (2) This paper is the first attempt to use bibliometric analysis which includes keyword co-occurrence analysis to classify AT-aided study status in ScienceDirect full-text database from 1946 to September 2021. VOSviewer, a tool for visualizing bibliometric graphs, is used for keyword co-occurrence analysis. It is able to gain insights into AT related topics through visualized keyword maps. With the help of VOSviewer, the systematically mixed quantitative and qualitative analysis summarizes the research categories, research hotspots, and research deficiencies of AT, which provide a reliable research method for the future study of AT. (3) This paper finds that the research status of AT-aided study primarily includes research methods, types, populations, diseases, therapeutic methods, and evaluation of six themes. The hot keywords of AT-aided health and well-being mainly focus on group AT, children, schizophrenia, well-being, mental health, palliative care, veterans, and the elderly, which may be used as a basis to analyze the background of the COVID-19 in the future AT-aided health and well-being. In addition, in spite of the gaps and shortcomings in the exploration of AT in behavior, movement, and technology, the integration and innovation of behavior, movement, and technology in the field of AT is a multidimensional breakthrough in promoting health and well-being. However, the research in this paper has certain limitations. Different researchers may use different keywords to express the same meaning due to differences in terms used by individuals, such as ‘older adults’ representing ‘the elderly’, which could affect the retrieval effect. In addition, VOSviewer software cannot automatically homogenize repeated synonymous keywords when performing keyword co-occurrence analysis. As such, manual homogenization of keywords may lead to slight deviations in data analysis. Further, this paper only conducts bibliometric analysis on a single ScienceDirect database, and does not extend the search data of AT to multiple databases in different fields such as health, society, and art. Future research could be based on the research status and research limitations of AT to systematically conduct visual analysis from multiple databases, such as Web of Science and Scopus, in order to create more application values for various social situations, such as COVID-19. Further, based on the research in the fields of behavior, movement, and technology, the therapeutic effect of AT could be analyzed more comprehensively and concretely from the aspect of promoting health and well-being.

## Figures and Tables

**Table 1:** High-frequency keywords of art therapy (AT)-aided health and well-being study from year 1946 to September 2021 via network visualization of VOSviewer (devised by the authors).

Words Color	Cluster	Keyword	Occurrences	Total Link Strength
	11	children	20	18.00
	2	depression	17	17.00
	10	trauma	17	16.00
	1	psychotherapy	16	16.00
	4	art	16	14.00
	3	cancer	16	13.00
	4	music therapy	13	13.00
	1	creativity	13	13.00
	2	drama therapy	10	10.00
	7	mental health	10	10.00
	5	breast cancer	10	10.00
	10	post-traumatic stress disorder (PTSD)	9	9.00
	3	stress	9	9.00
	3	quality of life	8	8.00
	9	palliative care	8	7.00
	8	computer system	8	5.00
	6	self-esteem	7	7.00
	2	prison	7	7.00
	1	psychiatry	7	6.00
	5	assessment	7	6.00
	5	schizophrenia	7	5.00
	6	veterans	6	6.00
	12	pain	6	6.00
	12	anxiety	6	6.00
	6	qualitative research	6	5.00
	5	group art therapy (AT)	6	4.00
	5	well-being	5	5.00
	1	Alzheimer’s disease	5	5.00
	11	epilepsy	5	5.00
	4	psychosis	5	5.00
	8	dementia	5	5.00
	10	military	5	5.00
	4	creative arts therapy (CAT)	5	4.00
	1	drawing	5	4.00
	4	dance/movement therapy (D/MT)	4	4.00
	11	adolescents	4	4.00

**Table 2:** Six thematic categories of AT-aided health and well-being (devised by the authors).

Thematic Category	Table 1 Associated Cluster	Keyword
Research methods of AT-aided study	6	qualitative research
Types of AT	4, 5	CAT, group AT
Research populations of AT	6, 10, 11	children, veterans, adolescent
AT-aided diseases	1, 2, 3, 4, 5, 8, 10, 11, 12	depression, trauma, cancer, breast cancer, PTSD, stress, schizophrenia, pain, anxiety, Alzheimer’s disease, epilepsy, psychosis, dementia
Therapeutic methods of AT	1, 2, 4, 9	psychotherapy, music therapy, drama therapy, palliative care, psychiatry, D/MT, drawing
Evaluation of AT-aided study	5, 8	computer system, assessment

**Table 3:** The list of creative arts therapy (CAT) studies for health and well-being (devised by the authors).

Source	Year	Research Method	Research Population	Research Disease	Therapeutic Method
Aldridge et al. [90]	1990	Case study	Art therapist	Epileptic	Painting and music
Milliken [91]	2002	Case study	Prisoners	Violence	Dance/movement
Talwar [92]	2007	Case study	Art therapist	PTSD	Dance, movement, music, poetry, and tempera
Brown [93]	2008	Experiment	Art therapist	None	Improvisations, music, sound, movement, writing, and art
Nordstrom-Loeb [94]	2012	Case study and questionnaire	General public	None	Dance/movement
van Westrhenen and Fritz [51]	2014	Literature review	Children	Trauma	None
Quinlan et al. [86]	2016	Experiment and questionnaire	Adolescents	Psychological and behavioral problems	Visual arts and play activities
Edwards [95]	2016	Literature review	General public	None	Music, art, and dance/movement
Papagiannaki and Shinebourne [96]	2016	Interview	Art therapist	Mental illness	None
Hertrampf and Wärja [64]	2017	Literature review	Adult women	Breast or gynecological cancer	Music, drama, dance/movement, and poetry
Levy et al. [89]	2018	Case study	Veterans	Mental health care and rehabilitation	Arts, dance/movement, and telehealth
Hanvey and Tepper-Lewis [33]	2019	Case study	Child and adolescent	Emergency psychiatry	Dance/movement and visual art
Spooner et al. [87]	2019	Case study	Veterans	Mental health care and rehabilitation	Visual art, dance/movement, music, and drawing
Hylton et al. [35]	2019	Experiment, questionnaire, interview	Adolescent	PTSD, depression, and anxiety	Visual art, drama, and music
Ali and Haen [88]	2019	Literature review	Veterans	Trauma	Dramatic, movement performance, music, and visual arts
Bechtel et al. [97]	2020	Case study	General public	None	Drama and tape sculpture

**Table 4:** The list of group AT studies for health and well-being (devised by the authors).

Source	Year	Research Method	Research Population	Research Disease	Therapeutic Method
Stone and Williams [104]	1982	Experiment and case study	Mothers of autistic children	Autistic	Drawing and discussion of the art work
Virshup [105]	1985	Case study	Drug abusers	Psychological problems	Drawing
Hagood [106]	1991	Case study	Mothers of sexually abused children	Psychological problems	Collage, drawing, and visual imagery
Rosal [107]	1993	Experiment	Children	Behavior disorder	Cognitive-behavioral approach
Liao and Liu [100]	2012	Experiment	Children	ADHD and emotional dysregulation	Drawing
Choi and Goo [98]	2012	Experiment, questionnaire, and case study	Mothers	Mother–child attachment	Drawing, collage, clay, and mask
Jang and Choi [108]	2012	Experiment and case study	Adolescents	Ego-resilience	Clay and pottery work
Kim et al. [103]	2014	Experiment	Adolescents	Depression and anxiety	Breath meditation and drawing
Stevenson et al. [109]	2014	Experiment	General public	Non-psychotic mental health disorders	Wait-list, art, and verbal therapy
Kim et al. [101]	2016	Experiment	Older patients	Neurocognitive disorders	Drawing and art materials
Korostiy and Hmain [110]	2016	Experiment	Patients	Recurrent depressive disorder	Drawing, clinical, psychopathological, psychodiagnostics, and statistical methods
Gabel and Robb [85]	2017	Thematic meta-synthesis	General public	None	None
Ünsalver and Sezen [111]	2017	Experiment and questionnaire	Pregnant women	Fear of giving birth	Psychoeducation, group AT
Kozhyna et al. [30]	2017	Experiment	Patients	Major depressive disorder	Drawing
Abdulah and Abdulla [40]	2018	Experiment	Children	Cancer	Painting and handcrafting
Sezen and Ünsalver [102]	2019	Experiment and questionnaire	Pregnant women	Fear of childbirth	Listening to music and singing, drawing, mask-making, mandala-making, puppet-making, taking photographs, and collage making
MacDonald et al. [43]	2019	Experiment, case study, and questionnaire	Youth and young adults	Diabetes	Drawing, collage and creating sculpture from paper, plasticine, self-hardening clay, fabrics or found objects
Teoli [112]	2021	Cooperative inquiry, companioning, and art-based research (interview)	Art therapists	Alzheimer’s, dementia, developmental disabilities, PTSD, anxiety, and psychological problems	Art making
Van Lith et al. [113]	2021	Experiment and questionnaire	Children	Mental health problems	Collage, drawing, and art materials (i.e., clay, materials)
Lee [99]	2021	Experiment and questionnaire	Mothers of children with disabilities	Parenting stress, perceived stress, depression, and perceived social support	Drawing and collage

**Table 5:** The list of studies for computer system in the evaluation of therapeutic effect on AT-aided health and well-being (devised by the authors).

Source	Year	Research Method	Research Population	Research Disease	Therapeutic Method
Hartwich and Brandecker [180]	1997	Case study	Inpatient	Borderline case personality disorder and psychotic diseases	Drawing and psychotherapy
Kim et al. [181]	2007	Case study	General public	None	Drawing
Kim et al. [182]	2008	Case study	General public	None	Drawing
Kim et al. [186]	2009	Experiment and case study	Old persons	Dementia	Structured mandalas
Mihailidis et al. [73]	2010	Participatory design (ethnography, questionnaire, and interview)	General public	Dementia	Visual arts, music, writing, painting, sculpture, and dance
Mattson [187]	2010	Literature review	General public	None	None
Kim et al. [185]	2011	Questionnaire and case study	General public	None	Kinetic family drawing
Mattson [184]	2011	Experiment	Middle-aged adults	Schizophrenia	Drawing (person picking an apple from a tree)
Kim et al. [183]	2012	Regression model and case study	Elderly people	Dementia	Painting (person picking an apple from a tree, face stimulus assessment, and structured mandala coloring)
Mattson [188]	2015	Usability evaluation (questionnaire and interview)	General public	None	Drawing
Kim et al. [101]	2016	Experiment	Older patients	Neurocognitive disorders	Drawing and art materials

## Data Availability

Publicly available datasets were analyzed in this study. These data can be found here: https://www.sciencedirect.com/ (accessed on 30 September 2021).
